# Protective Effect of *Artocarpus heterophyllus* Lam. (Jackfruit) Polysaccharides on Liver Injury Induced by Cyclophosphamide in Mice

**DOI:** 10.3390/nu16010166

**Published:** 2024-01-04

**Authors:** Ming Cheng, Yifan Zheng, Gang Wu, Lehe Tan, Fei Xu, Yanjun Zhang, Xiaoai Chen, Kexue Zhu

**Affiliations:** 1 Spice and Beverage Research Institute, Chinese Academy of Tropical Agricultural Sciences, Wanning 571533, China; 2 School of Food Science and Engineering, Hainan University, Haikou 570228, China; 3 College of Food Science and Engineering, Jilin Agricultural University, Changchun 130118, China; 4 National Center of Important Tropical Crops Engineering and Technology Research, Wanning 571533, China; 5 Key Laboratory of Processing Suitability and Quality Control of the Special Tropical Crops of Hainan Province, Wanning 571533, China

**Keywords:** jackfruit polysaccharides, liver injury, protective effects, metabolomics

## Abstract

In recent years, *Artocarpus heterophyllus* Lam. (jackfruit) polysaccharides (namely JFP-Ps) have attracted much attention due to their multiple biological activities. This study aimed to explore the protective effects and the underlying mechanisms of JFP-Ps on cyclophosphamide (Cp)-induced liver damage. The protective effect of JFP-Ps was evaluated using HE staining, antioxidant testing, enzyme-linked immunosorbent assay (ELISA), real-time quantitative polymerase chain reaction (RT-qPCR), Western blot and ultra-performance liquid chromatography equipped with quadrupole time-of-flight mass spectrometry (UPLC-Q-TOF-MS/MS) metabolomics analysis. The results showed that Cp caused pathological liver damage, activated oxidative stress and downregulated cytokine expression, while JFP-Ps treatment was found to exert antioxidant effects and play immune regulatory roles through mitogen-activated protein kinase/nuclear factor-κB (MAPK/NF-κB) related inflammation and cell apoptosis pathways to protect the Cp-induced liver injury. Metabolomic results showed that the liver-protective effects of JFP-Ps were mainly related to aminoacyl transfer ribonucleic acid (tRNA) biosynthesis, sphingolipid metabolism, purine metabolism and the citrate cycle. These results indicate that JFP-Ps have great potential application in alleviating liver injury.

## 1. Introduction

The liver is an important metabolic organ and is responsible for the synthesis, decomposition, transformation and excretion of many molecules and other metabolic processes [1]. Therefore, the liver is the main target of drugs and toxins and is damaged through drug-associated adverse effects [2]. Cyclophosphamide (Cp), a cell cycle non-specific drug, is widely used in antitumor therapy due to its ability to inhibit the proliferation of cancer cells [3]. However, Cp is hepatotoxic and can damage normal hepatocytes. It has been reported that Cp can be metabolized into active substances, such as phosphoramidic mustard and acrolein, under the action of cytochrome P450, which catalyzes the alkylation reaction between these metabolites and the guanine base of DNA [4], inhibiting the synthesis of DNA and RNA and causing DNA damage, leading to oxidative stress and cytotoxic effects [5].

Polysaccharides are natural polymer compounds that are widespread in animals, plants and microorganisms. Some exhibited antioxidant, anti-inflammatory, immune regulation and liver protection activities and have become a research hotspot in various fields [6,7]. Jackfruit is a tropical fruit native to the Western Ghats in India and has been introduced and cultivated in tropical and subtropical countries, such as Bangladesh, Myanmar, Sri Lanka, Malaysia, Indonesia, Philippines and Thailand. It was imported to China thousands of years ago and has become one of the commercial crops cultivated in Hainan, Guangdong, Guangxi, Fujian and Taiwan provinces [8]. Our research group previously purified a polysaccharide from *Artocarpus heterophyllus* Lam. (jackfruit) pulp (JFP-Ps), with a molecular weight of 1668 kDa and strong antioxidant activity [9]. It has been reported that JFP-Ps increased the abundance of beneficial gut bacteria and restored the gut microbiota of obese rats, which may have an impact on health [10]. In addition, JFP-Ps activated the peroxisome proliferator activated receptor (PPAR) and adenosine monophosphate-activated protein kinase (AMPK) signaling pathways to alleviate non-alcoholic fatty liver disease [11].

At present, little is known about the effect and mechanism of JFP-Ps on liver injury. Therefore, this work aimed to evaluate the hepatoprotective effect of JFP-Ps against cp-induced liver injury in mice. A metabolomics method based on ultra-performance liquid chromatography equipped with quadrupole time-of-flight mass spectrometry (UPLC-Q-TOF-MS/MS) was used to identify endogenous metabolites and related metabolic pathways. This study provides a theoretical basis for the development of JFP-Ps as a potential natural compound for the treatment of liver injury.

## 2. Materials and Methods

### 2.1. Materials and Reagents

The JFP-Ps were extracted, purified and prepared according to our previous method at the Spice and Beverage Research Institute, Chinese Academy of Tropical Agricultural Sciences [9].

Malonic dialdehyde (MDA), superoxide dismutase (SOD), catalase (CAT) and glutathione peroxidase (GSH-Px) assay kits were purchased from Suzhou Geruisi Biotechnology Co., Ltd. (Suzhou, China). Tumor necrosis factor alpha (TNF-α), interleukin-2 (IL-2), interleukin-6 (IL-6), interleukin-10 (IL-10) and interferon gamma (IFN-γ) assay kits were purchased from Shanghai Enzyme-linked Biology Co., Ltd. (Shanghai, China). Primers were purchased from Shenggong Biotechnology Co., Ltd. (Shanghai, China). Polyclonal antibodies of β-actin (20536-1-AP), IκBα (10268-1-AP), nuclear factor kappa-B (NF-κB) p65(10745-1-AP), p38 MAPK (14064-1-AP), JNK (24164-1-AP) and anti-rabbit IgG (SA00001-2) were purchased from Proteintech Group, Inc. (Wuhan, China). Phospho-p38 MAPK (p-p38, AP0526) was purchased from ABclonal, Inc. (Wuhan, China), phospho-JNK (p-JNK, ab76572) was purchased from Abcam (Shanghai) Trading Co., Ltd. (Shanghai, China). Phospho-NF-κB p65 (p-p65, AF5875) and polyvinylidene fluoride (PVDF) membrane were purchased from Beyotime Biotechnology Co., Ltd. (Shanghai, China).

### 2.2. Animal Experimental Design

The experimental animals were kept in accordance with the National Guidelines for Experimental Animal Care and Use, and the procedure was approved by the Animal Ethical Committee of Hainan Medical University (Permit # HYLL-2023-462). Fifty male BALB/C mice were supplied by Hunan Slac Laboratory Animal Co. Ltd. (Changsha, China), with the certificate number SCXK (Xiang) 2019–0004. Mice were fed with sufficient chow and water in a laboratory environment for acclimatization. After adaptation for 1 week, 40 mice were selected to induce liver injury by Cp using intraperitoneal injection, and the remaining 10 mice were given physiological saline as the normal control group (NC). After three days, the 40 Cp-induced liver injury mice were equally separated into four groups. (1) Model control group (MC): given physiological saline; (2) JFP-Ps low-dose group (LG): 50 mg JFP-Ps/kg body weight; (3) JFP-Ps medium-dose group (MG): 100 mg JFP-Ps/kg body weight; (4) JFP-Ps high-dose group (HG): 200 mg JFP-Ps/kg body weight. After JFP-Ps intervention for one week, all mice were anesthetized with 5% chloral hydrate after 12 h of fasting, and then sacrificed via cervical dislocation.

### 2.3. Histopathological Observation

The liver samples were fixed in 4% paraformaldehyde and then dehydrated with alcohol, embedded in paraffin, cut into 4 μm slices and stained with hematoxylin and eosin (H&E). The samples were observed under a light microscope (Nikon, Tokyo, Japan) and photographed.

### 2.4. Determination of Biochemical Indicators

Liver samples were homogenized in phosphate-buffered saline (PBS, pH 7.4) and centrifuged at 2500× *g* for 20 min to collect supernatants for further determination of the contents of MDA, TNF-α, IL-6, IL-2 and IFN-γ, as well as the activities of SOD, CAT and GSH-Px, according to the manufacturer’s instructions.

### 2.5. Real-Time Quantitative PCR

Total RNA in the liver was extracted with Trizol and cDNA was prepared using BeyoRT^TM^ III first-strand synthesis kit (Beyotime Biotechnology Co., Ltd., Shanghai, China). A three-step approach was used for target genes amplification. Relative mRNA expression level was calculated using the 2^−ΔΔCt^ method, with β-actin used for normalization. The primer information is listed in Table 1.

### 2.6. Western Blot

Liver sample were homogenized and centrifuged at 12,000× *g*, 4 °C for 10 min in 4 °C to collect supernatants. The protein concentration in the supernatants was measured and adjusted to 3.5 μg/μL. Equal amounts of denatured proteins were separated using 12% SDS-PAGE and transferred onto a 0.45 µm PVDF membrane. Then, the PVDF membrane was blocked with 5% skim milk and incubated with primary antibodies. Lastly, the PVDF membrane was incubated with the secondary antibody for an hour to photograph the band. The antibodies used were β-actin (1:2000), IκB α (1:2000), p65 (1:2000), p-p65 (1:1000), p38 (1:1000), p-p38 (1:2000), JNK (1:2000), p-JNK (1:5000) and secondary antibodies (anti-rabbit IgG, 1:2000). The gray value was measured using Image J.

### 2.7. UPLC-Q-TOF-MS/MS Analysis

Liver samples were homogenized in methanol, ethanol and distilled water at a ratio of 2:2:1 and ultrasonicated for 10 min. The resulting mixture was centrifuged at 12,000× *g*, 4 °C for 15 min, and the supernatant was collected. The sample (3 μL) was separated on an Agilent 1290 ultra-high performance liquid chromatography system using Eclipse Plus C18 at 35 °C with 0.1% formic acid solution (mobile phase A) and acetonitrile (mobile phase B) at a flow rate of 0.4 mL/min. A 35 min gradient program was set as follows: 0–1.5 min 5% B, 1.5–15 min 5–60% B, 15–25 min 60–100% B, 25–30 min 100% B, 30–30 min 100–5% B, and 30–35 min 5% B.

Mass spectrometry (MS) data were obtained using an Agilent 6530B Q-TOF mass spectrometer (Agilent Technologies, Santa Clara, CA, USA) in positive and negative electrospray ionization (ESI) modes. The drying gas temperature was 325 °C; wavelength was 250 nm; cone voltage was 65 V; attenuation was 1000 mAU; draw speed was 100 μL/min; eject speed was 400 μL/min; MS scan range was from 50 to 1200 *m*/*z*; primary mass spectrometry scan was 2 spectra/s and MS scan time was 4 spectra/s.

### 2.8. Statistical Analysis

Results are presented as the mean ± SEM. Data were analyzed using one-way ANOVA followed by Duncan’s multiple range test using SPSS Statistics 26 (IBM Inc., Armonk, New York, NY, USA). A *p* value < 0.05 indicated a significant difference.

The UPLC-Q-TOF-MS data were collected using Masshunter (Agilent Technologies, Inc.), processed using Profinder (Agilent Technologies, Inc.) and normalized and filtered using Mass Profiler Professional (MPP, Agilent Technologies, Inc.). Principal component analysis (PCA) and orthogonal partial least-squares discriminant analysis (OPLS-DA) were used to identify the differences in metabolites of different groups based on FC (Fold change) > 2 and *p* < 0.05. The identified metabolites were determined through searching databases and confirmed using UPLC-Q-TOF-MS/MS.

## 3. Results

### 3.1. Effects of JFP-Ps on the Histopathology of the Liver

**Figure 1:**
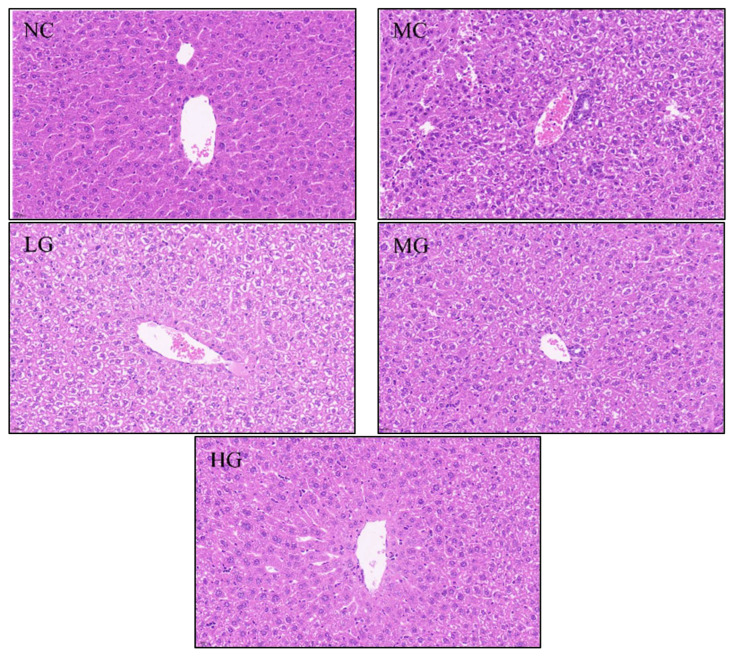
Effects of JFP-Ps on liver histopathological characteristics (original magnification 40×, bar 20 μm). NC: normal control group; MC: model control group; LG: JFP-Ps low-dose group at 50 mg JFP-Ps/kg body weight; MG: JFP-Ps medium-dose group at 100 mg JFP-Ps/kg body weight; HG: JFP-Ps high-dose group at 200 mg JFP-Ps/kg body weight.

Histopathological examination shows that Cp causes liver damage. As shown in Figure 1, the hepatocytes in the NC group show normal histological structure. However, in the MC group, hepatocytes are scattered and structurally abnormal, with necrotic hepatocytes having small fragmented nuclei and inflammatory-infiltrating hepatocytes. A large number of fat droplets were deposited in the portal vein, and the small spaces in the portal vein became congested and inflamed. After treatment with different doses of JFP-Ps, the morphology and structure of the liver cells were alleviated to varying degrees compared to those of the MC group. Inflammation and cavitation were restored, and portal vein fat deposition was reduced.

### 3.2. Effect of JFP-Ps on Oxidative Stress in the Liver

The effects of JFP-Ps on oxidative stress were studied through measuring the level of MDA and the activities of SOD, CAT and GSH-Px in the liver homogenate. As shown in Table 2, compared with the NC group, the content of MDA in the MC group was significantly increased (*p* < 0.05), while the activities of SOD, CAT and GSH-Px were significantly decreased (*p* < 0.01). The contents of MDA in the liver homogenate of the JFP-Ps groups were lower than those of the MC group, but there were no significant differences (*p* > 0.05). The activities of SOD, CAT and GSH-Px tended to increase compared to those in the MC group (*p* < 0.05). These results suggest that JFP-Ps may regulate oxidative stress responses and attenuate liver damage.

### 3.3. Effect of JFP-Ps on the Level of Cytokines in the Liver

The level of cytokines in the liver homogenate was measured using ELISA. Compared with the NC group, the level of IL-6 was significantly decreased in the MC group (*p* < 0.05) and the levels of IL-2 and TNF-α also decreased in the MC group, but there was no significant difference (*p* > 0.05, Table 3). The level of IFN-γ in the MC group was higher than that in the NC group (*p* < 0.05). After intervention with different doses of JFP-Ps, the levels of IL-2, IL-6, TNF-α and IFN-γ were reversed to varying degrees compared with those in the MC group.

### 3.4. Effect of JFP-Ps on mRNA Expression of Inflammation-Related Genes in the Liver

**Figure 2:**
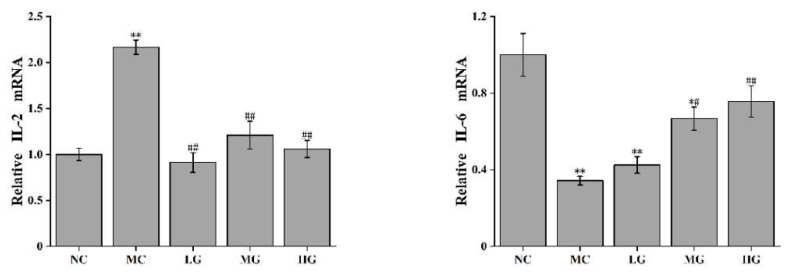

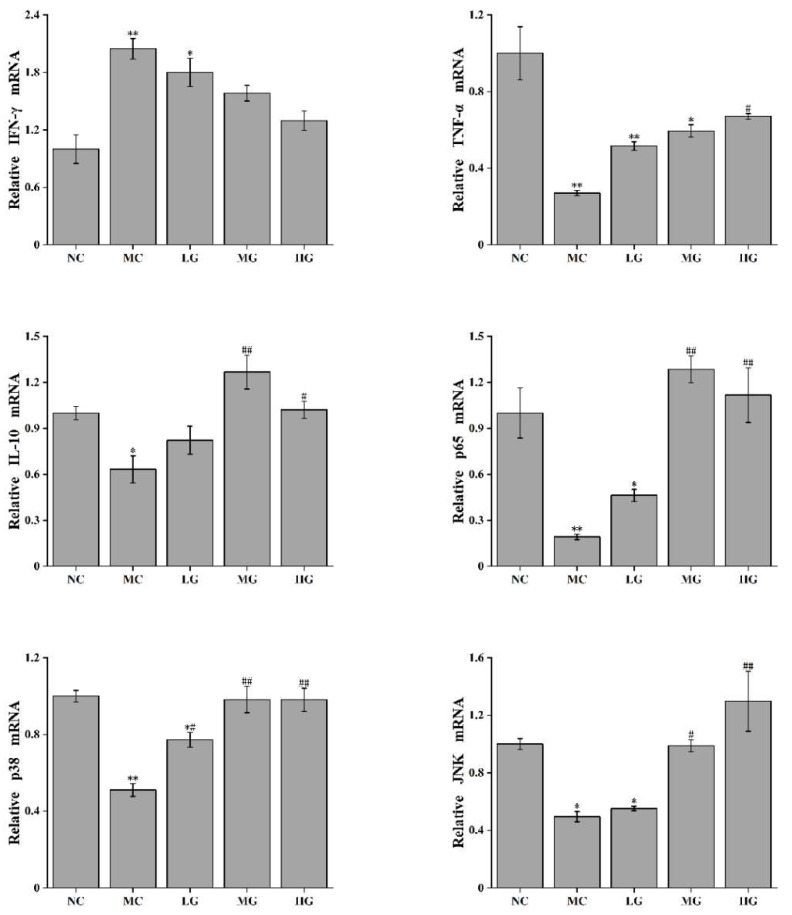
Effect of JFP-Ps on the mRNA expression of inflammation-related genes in the liver. Compared with NC group: * *p* < 0.05, ** *p* < 0.01; Compared with MC group: ^#^ *p* < 0.05, ^##^ *p* < 0.01. NC: normal control group; MC: model control group; LG: JFP-Ps low-dose group at 50 mg JFP-Ps/kg body weight; MG: JFP-Ps medium-dose group at 100 mg JFP-Ps/kg body weight; HG: JFP-Ps high-dose group at 200 mg JFP-Ps/kg body weight.

As shown in Figure 2, compared with the NC group, the expression levels of IFN-γ and IL-2 mRNA in the MC group were significantly increased (*p* < 0.01), while the expression levels of TNF-α, IL-6 and IL-10 mRNA were significantly reduced (*p* < 0.05). After treatment with JFP-Ps at different doses, the mRNA expression levels of IL-2 and IFN-γ were significantly decreased compared to those in the MC group (*p* < 0.05), while the mRNA expression levels of TNF-α, IL-6 and IL-10 were significantly increased compared to those in the MC group (*p* < 0.05). The increased expression of IFN-γ and IL-2 mRNA and the decreased expression of IL-10 mRNA in the MC group indicate that liver tissue may be damaged via inflammation.

In addition, the mRNA expression levels of p65, p38 and JNK proteins were measured. The results showed that their mRNA expression levels in the MC group were significantly downregulated (*p* < 0.05) compared with those in the NC group. After the JFP-Ps intervention, the mRNA expression levels were restored compared to those in the MC group (*p* < 0.05).

### 3.5. Effect of JFP-Ps on Protein Expression of the Proteins Involved in Inflammation-Related Pathways in the Liver

**Figure 3:**
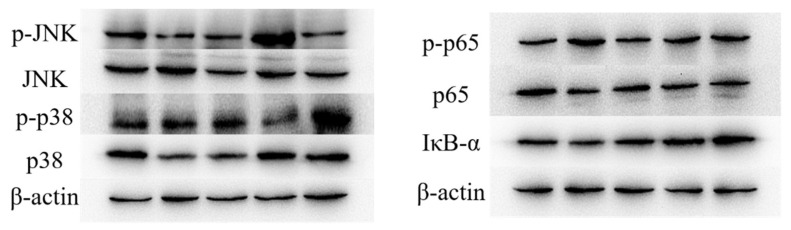

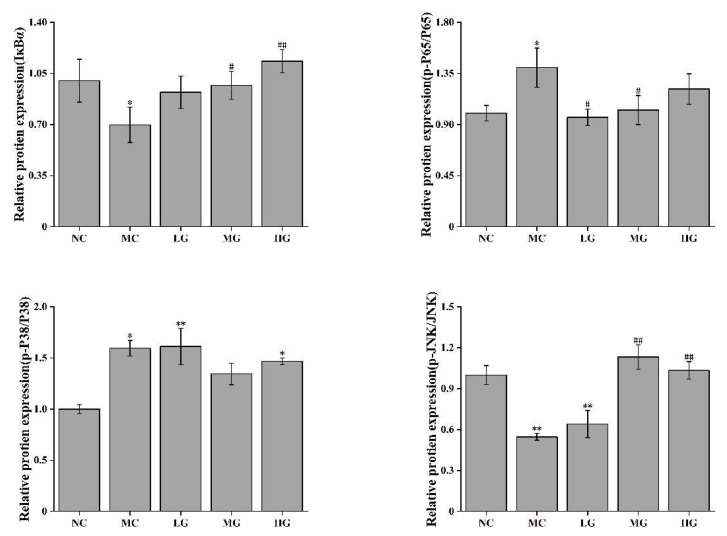
Effect of JFP-Ps on protien expression of the proteins involved in inflammation-related pathways. Compared with NC group: * *p* < 0.05, ** *p* < 0.01; Compared with MC group: ^#^ *p* < 0.05, ^##^ *p* < 0.01. NC: normal control group; MC: model control group; LG: JFP-Ps low-dose group at 50 mg JFP-Ps/kg body weight; MG: JFP-Ps medium-dose group at 100 mg JFP-Ps/kg body weight; HG: JFP-Ps high-dose group at 200 mg JFP-Ps/kg body weight.

The results of the Western blot showed that JFP-Ps intervention downregulated the expression levels of p-p65/p65 protein and phosphorylated p-p38/p38 protein (*p* < 0.05), while upregulating IκB-α protein and p-JNK/JNK protein expression levels (*p* < 0.05) (Figure 3). These results indicate that JFP-Ps can regulate the MAPK apoptosis pathway and inhibit the NF-κB/p65 inflammatory pathway to protect the liver.

### 3.6. Metabolic Profile of UPLC-Q-TOF-MS in the Liver

**Figure 4:**
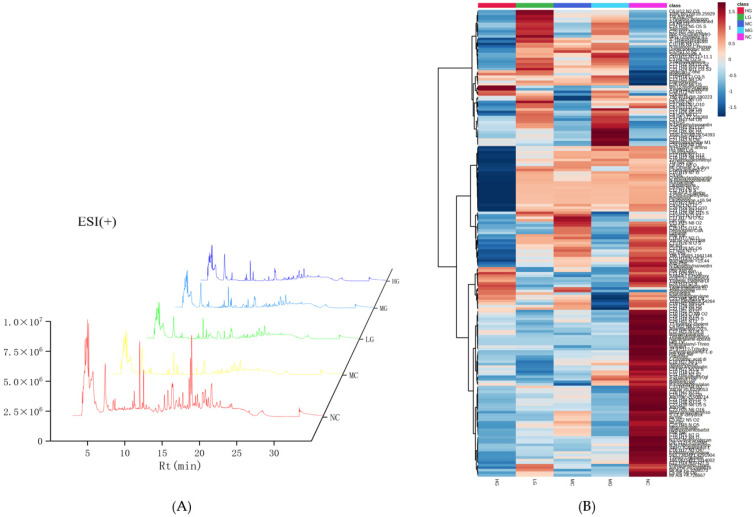

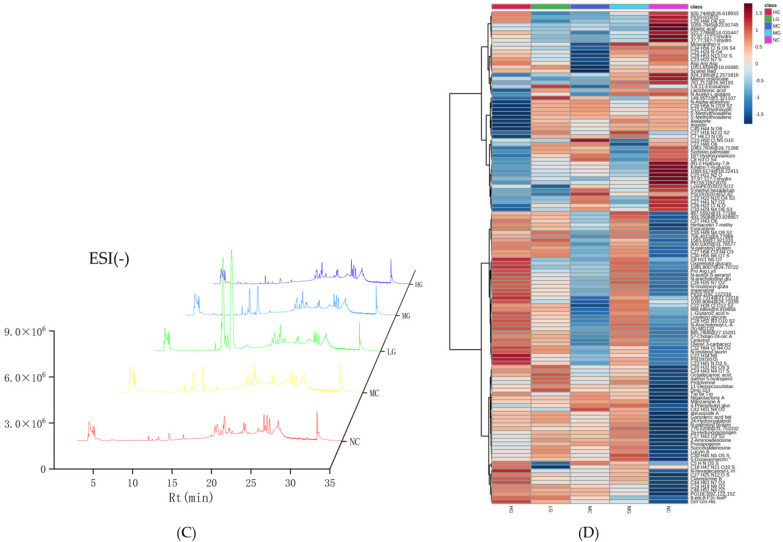
Total Ion Chromatogram (TIC) map and metabolites heatmap in positive (**A**,**B**) and negative (**C**,**D**) electrospray ionization (ESI) modes. NC: normal control group; MC: model control group; LG: JFP-Ps low-dose group at 50 mg JFP-Ps/kg body weight; MG: JFP-Ps medium-dose group at 100 mg JFP-Ps/kg body weight; HG: JFP-Ps high-dose group at 200 mg JFP-Ps/kg body weight.

UPLC-Q-TOF-MS was used to collect data from the liver extract (Figure 4). The peak numbers, positions, and intensities of the five different treatment groups showed significant differences, indicating significant changes in the types and quantities of metabolites in the liver samples from different groups. Quality control was conducted on the obtained primary data using *t*-test (Table 4). A total of 227 substances were found in the positive mode (ESI+) (*p* < 0.05), while 398 substances were found in the negative mode (ESI−) (*p* < 0.05). The contents of these substances in the treatment groups were visualized using heat maps.

### 3.7. Multivariate Statistical Analysis

**Figure 5:**
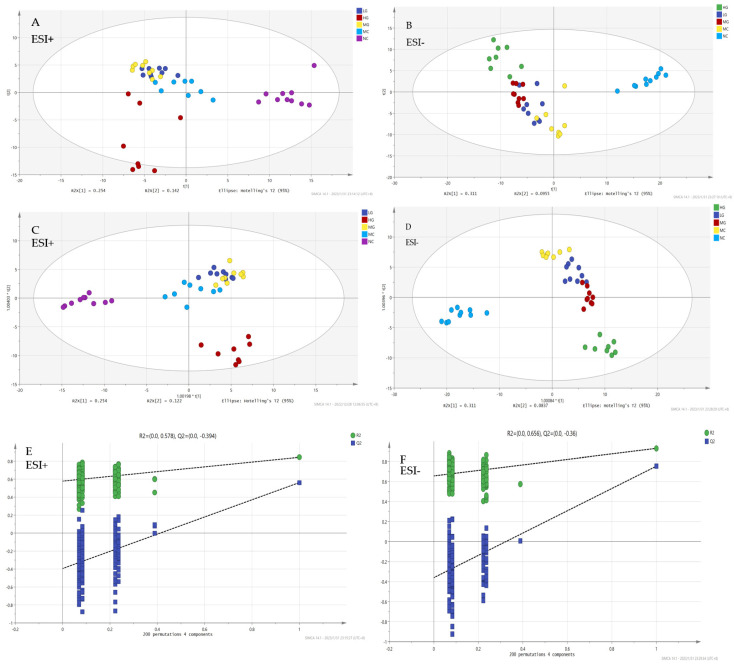
Multivariate statistical analysis ((**A**,**B**) PCA score map; (**C**,**D**) OPLS-DA score map; (**E**,**F**) 200 permutation test).

The PCA and OPLS-DA showed good intergroup differences and intragroup clustering among liver metabolites in different treatment groups (Figure 5). The R^2^ and Q^2^ values of the PCA model in ESI+ were 0.544 and 0.335, respectively, while the R^2^X, R^2^Y and Q^2^ values of the OPLS-DA model were 0.527, 0.894 and 0.718, respectively. The R^2^ and Q^2^ values of the PCA model in ESI− were 0.531 and 0.345, respectively, while the R^2^X, R^2^Y and Q^2^ values of the OPLS-DA model were 0.51, 0.938 and 0.75, respectively. The results suggest that the interpretability and predictability of the model were acceptable. The OPLS-DA model was tested for 200 times using displacement tests. The R^2^Y and Q^2^Y values of ESI+ were 0.576 and −0.394, respectively, and the R^2^Y and Q^2^Y values of ESI− were 0.656 and −0.36, respectively, indicating that the model did not overfit and the results are reliable. The PCA maps show a good separation between the NC group and the MC group.

To make the results more reliable, OPLS-DA was used for analysis. As shown in the OPLS-DA map, although the separation between the LG and MG groups and the MC group was still not significant in ESI+, there were significant intergroup differences and intragroup clustering in ESI−. These results indicate that there are certain differences in metabolites among the different groups. The metabolites in the liver under the Cp induced liver injury group were different from those in the normal liver group and could be regulated through JFP-Ps intervention. High doses of JFP-Ps may have a significant impact on the changes in these liver metabolites.

### 3.8. Identification of Metabolites

**Figure 6:**
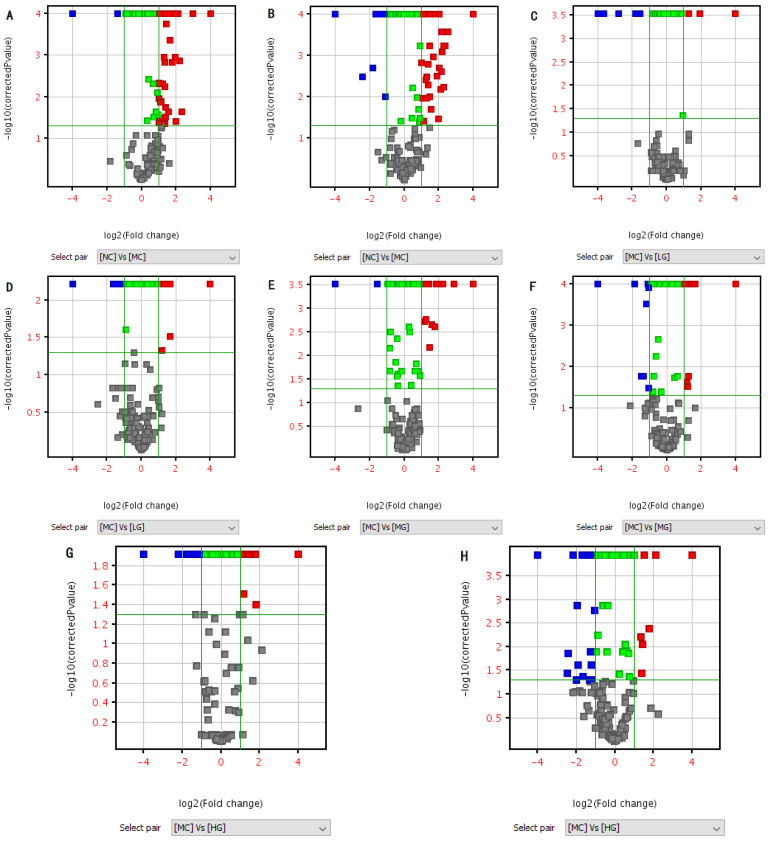

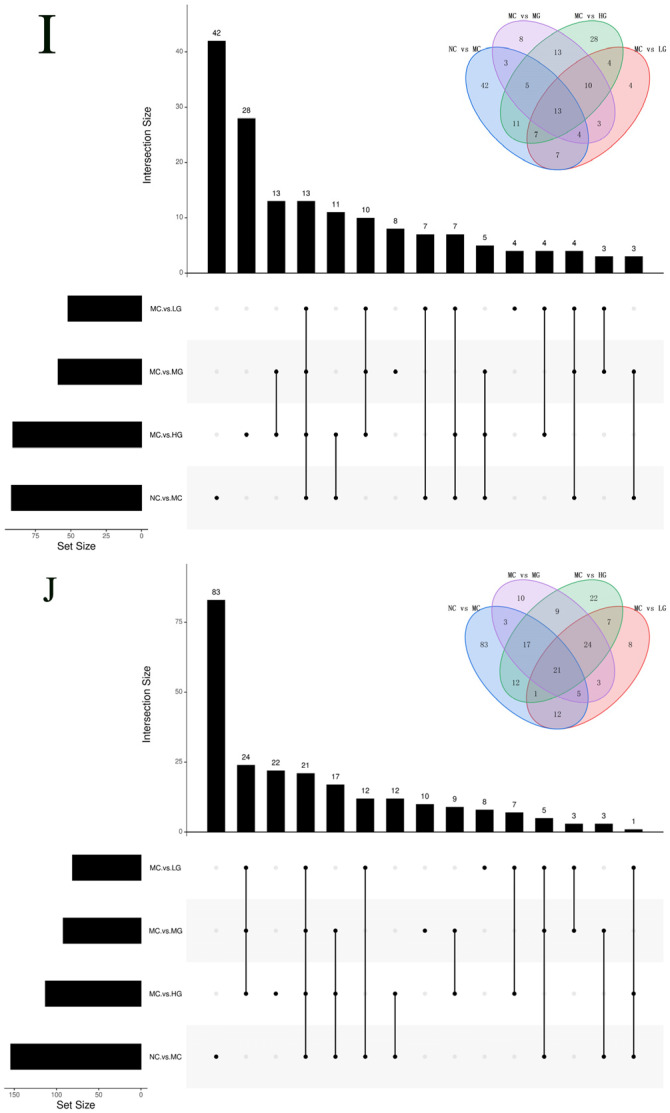
Volcano map ((**A**): NC vs. MC in ESI+, (**B**): NC vs. MC in ESI−, (**C**): MC vs. LG in ESI+, (**D**): MC vs. LG in ESI−, (**E**): MC vs. MG in ESI+, (**F**): MC vs. MG in ESI−, (**G**): MC vs. HG in ESI+, (**H**): MC vs. HG in ESI−) and Veen map ((**I**) ESI+; (**J**) ESI−). Red: Metabolites which pass both log2 Fold Change > 1.0 and *p* < 0.05 cut-offs and are up-regulated. Blue: Metabolites which pass both log2 Fold Change < −1.0 and *p* < 0.05 cut-offs and are down-regulated. Green: Metabolites which pass the *p* < 0.05 cut-off and fail to pass the |log2 Fold Change| > 1.0 cut-off. Grey: Metabolites which neither pass the *p* < 0.05 cut-off nor the |log2 Fold Change| > 1.0 cut-off.

All detected metabolites were screened for differential metabolites based on FC (Fold change) > 2 and *p* < 0.05 (Figure 6). It was found that there were 92 differential metabolites in ESI+ and 154 in ESI− between the NC and MC groups. There were 52 differential compounds in ESI+ and 81 in ESI− between the MC and LG groups, 59 differential compounds in ESI+ and 92 in ESI− between the MC and MG groups and 91 differential compounds in ESI+ and 113 in ESI− between the MC and HG groups. Through analyzing these differential compounds using a Veen map, it was found that there were seven common differentials in each group in ESI+, 42 unique differentials in NC vs. MC, four unique differentials in MC vs. LG, eight unique differentials in MC vs. MG and 28 unique differentials in MC vs. HG; In ESI−, there were 12 common differences in each group, 83 unique differences in NC vs. MC, eight unique differences in MC vs. LG, 10 unique differences in MC vs. MG and 22 unique differences in MC vs. HG. These results indicate that the liver metabolites are significantly altered in Cp-induced liver injury mice, and JFP-Ps intervention can improve metabolic disorders in the liver caused by Cp.

The MS/MS information was extracted, and the differential metabolites were further confirmed using the METLIN and HMDB databases. A total of 31 differential metabolites were identified in the liver of mice with Cp-induced liver injury in both ESI+ and ESI− (Table 5 and Table 6). These substances mainly include amino acids, spermidine, niacinamide, xanthine, hypoxanthine, adenine, deoxyguanosine, glutathione, sphinganine, sphingosine, phytosphingosine, glucosylceramide, indole-3-carboxaldehyde and succinic acid.

### 3.9. Metabolic Pathway Analysis

**Figure 7:**
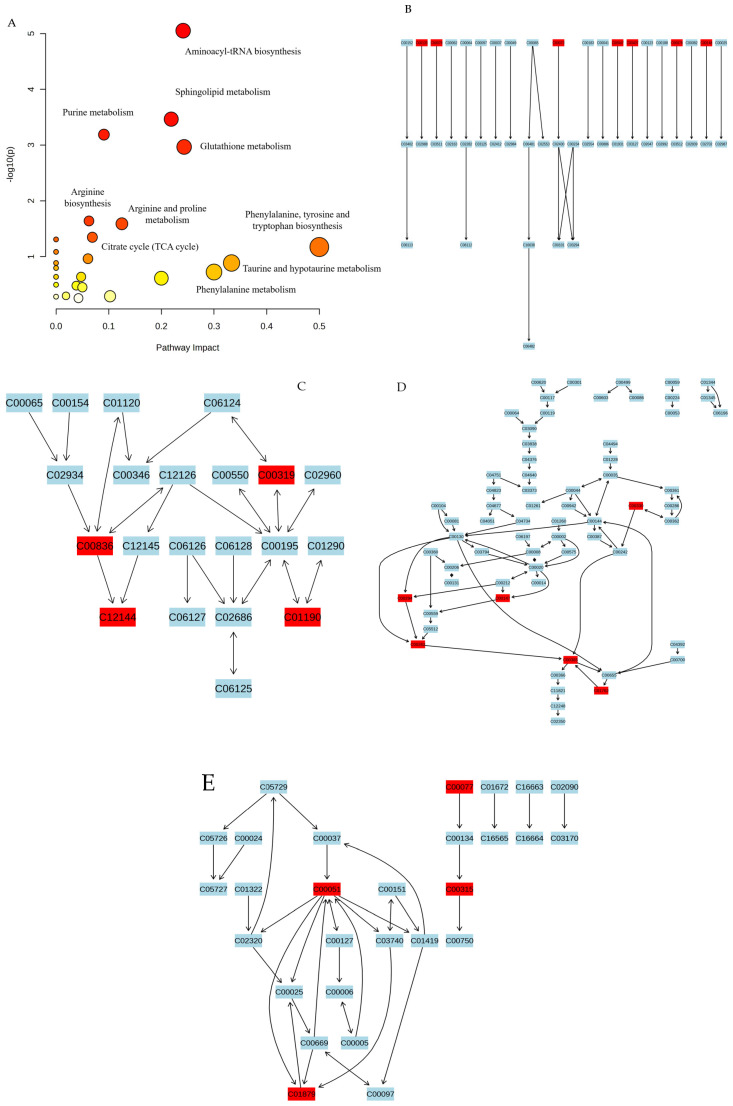
Pathway analysis. (**A**) metabolic pathway diagram; (**B**) Aminoacyl-tRNA biosynthesis; (**C**) Sphingolipid metabolism; (**D**) Purine metabolism; (**E**) Glutathione metabolism. The colors from yellow to red represent metabolites with different level of significance, red being more significant and yellow less significant respectively. The compounds highlighted in red font represent the metabolic markers in the metabolic pathways.

Metabolic pathway analysis was conducted on the screened differential metabolites. As shown in Figure 7 and Table 7, the metabolic pathways in the liver related to JFP-Ps intervention in Cp-induced liver injury mice mainly include aminoacyl tRNA biosynthesis, sphingolipid metabolism, purine metabolism, glutathione metabolism, arginine biosynthesis, arginine and proline metabolism and the citrate cycle.

## 4. Discussion

Cp induced hepatotoxicity including liver necrosis, inflammation and oxidative damage. MDA is the final product of lipid peroxidation; SOD is an antioxidant enzyme that can promote the disproportionation of superoxide into hydrogen peroxide to resist oxygen free radicals; and GSH-Px and CAT can protect cell structure and membrane function via catalyzing the decomposition of hydrogen peroxide [12]. The present study showed that the MDA level in the MC group was increased, while the activities of SOD, CAT and GSH-Px were decreased. However, the oxidative stress indicators in the JFP-Ps groups were alleviated and approached those of the NC group, which may be related to the free radical scavenging activity of JFP-Ps.

The inflammatory cytokines reflect the immune state to a certain extent and play an essential role in the immune response [13]. TNF-α is a multifunctional cytokine that stimulates the expression of a series of inflammatory mediators to regulate inflammation [14]. IFN-γ is mainly secreted by natural killer cells and has antiviral, immune regulation and other active functions [15]. IL-2 is a necessary cytokine for T cell proliferation and has an inductive effect on cells and memory cell generation [16]. IL-6 plays a key regulatory function in immunity and is involved in the development, maturation, and sustained antibody production of B cells [17]. It has been reported that *Lycium ruthenicum* Murr. polysaccharide enhanced serum cytokine expression in immunocompromised mice [18], which is consistent with our results, suggesting that JFP-Ps may exert regulatory effects on inflammation and immune function through regulating the secretion of these cytokines in the liver of Cp-exposed mice.

Liver damage is induced via the release of inflammatory cytokines, activation of apoptosis pathway, etc. [19]. The NF-κB signaling pathway is closely related to immunity, inflammation and cell apoptosis [20]. Cui et al. [21] reported that the polysaccharides from *Caulis spatholobi* had a protective effect mainly through regulating NF-κB signaling pathway in the intestinal mucosa of Cp-exposed chickens. The MAPK family, including ERK, JNK and p38, plays an important role in regulating cell apoptosis, cell cycle, cell growth inhibition and differentiation, as well as in mediating autophagy [22]. The activation of p38 signaling has been shown to induce the expression of pro-inflammatory cytokines, while JNK signaling under stress stimulation can induce inflammation or cell apoptosis [23]. The present study found that the liver damage was associated with the degradation of IκB-α protein and activation of the NF-κB inflammatory pathway, while JFP-Ps exerted a protective effect on the liver through inhibiting the p-p65/p65 and p-p38/p38 pathways and activating the p-JNK/JNK pathway.

Aminoacyl tRNA is the substrate for translation and is synthesized via matching amino acids with tRNA containing corresponding anticodons through aminoacyl tRNA synthase (ARS) [24]. ARS participates in translation and serves as a signaling molecule in the development of immune cells in various immune diseases to mediate immune responses [25]. Inflammatory signals can induce the phosphorylation of ARS and regulate the cascade reaction of various cytokines and MAPK and other cellular signaling pathways [26]. *Ascophyllum nodosum* polysaccharide has been reported to inhibit the progression of inflammation mainly through regulating inflammation-related signals, including phenylalanine, tryptophan biosynthesis, and aminoacyl tRNA biosynthesis [27]. Consistent with a previous study, JFP-Ps treatments altered the metabolism of various amino acids, including histidine, phenylalanine, methionine, isoleucine, lysine, proline and tryptophan, which are mainly related to the aminoacyl tRNA biosynthesis.

Arginine serves as a component of protein synthesis, and acts as a metabolic substrate for immune cells [28]. Arginine can be metabolized to nitric oxide (NO) by nitric oxide synthase (NOS), and the abnormal synthesis of NO can induce tissue damage, while arginase could act as a competitive enzyme to compete with arginine to prevent the generation of NO [29]. Arginine can modulate innate immune responses through modulating the MAPK signaling pathway [30]. Our results show that the metabolic product of arginine biosynthesis is imbalanced in the liver of Cp-exposed mice.

Sphingolipids are common components of eukaryotic cell membranes. Extracellular stimuli, such as cytokines and cellular stress, can disrupt normal cell homeostasis and disrupt sphingolipid metabolism. Ceramide and sphingosine are metabolites of sphingolipids that act as key signaling molecules in immunity and inflammation [31]. Changes in ceramide level may affect the interaction between lipids and proteins within the membrane, thereby affecting the transmission of intracellular signals [32]. Polysaccharides obtained from *Suanzaoren* decoction have been reported to reduce the concentrations of phytosphingosine, sphingosine and ceramide, inducing neuronal cell death as a mechanism of immune deficiency [33]. An abnormal sphingolipid metabolism was found in Cp-exposed mice, and JFP-Ps attenuated the expression of sphingosine, sphinganine, phytosphingosine and ceramide in the present study.

In general, purine nucleotides are regenerated through recycling pathways by hypoxanthine guanine phosphoribosyltransferase and adenine phosphoribosyltransferase [34]. Hypoxanthine can be oxidized by xanthine oxidase to form xanthine and guanine, which in turn can respectively generate hypoxanthine mononucleotide and guanylate to fulfill the purine requirements within cells [35]. We analyzed the metabolites (xanthine, hypoxanthine, adenine, guanosine and inosine) involved in purine metabolism. It was confirmed that Cp treatment caused oxidative damage to mouse liver, leading to an imbalance in purine metabolism, and JFP-Ps were beneficial for restoring this damage.

Glutathione (GSH) is a tripeptide mainly synthesized in the liver and acts as an antioxidant [36]. In addition, GSH is essential for activating T lymphocytes and multiple signaling pathways, including NF-κB, p38 and JNK [37]. The citric acid (TCA) cycle has emerged as an energy metabolism hub and a regulator of immune responses in most eukaryotes. Mitochondria are critically involved in cell proliferation, death, differentiation and immunity via driving macrophage polarization through IL-6, and via activating mitochondrial signaling through inflammatory factors like TNF-α [38]. Succinate, which is formed in the TCA cycle, accumulates under inflammatory or stress conditions, and is involved in macrophage activation [39]. Fumarate is considered anti-inflammatory, and its degradation is detrimental to the host and abrogates trained immunity [39]. In the present study, JFP-PS attenuated liver injury, which may be associated with the TCA cycle.

## 5. Conclusions

JFP-Ps have a hepatoprotective effect on Cp-induced liver injury via inhibiting peroxidative damage and regulating the expression of related genes. UPLC-Q/TOF-MS/MS-based metabolomics results showed that the liver-protective effects of JFP-Ps were mainly related to aminoacyl tRNA biosynthesis, sphingolipid metabolism, purine metabolism, glutathione metabolism, arginine biosynthesis, arginine and proline metabolism and the citric acid cycle. The results of this Cp model indicate that JFP-Ps may have the potential to alleviate liver injury. Whether this effect can be transferred to other liver noxae and whether it can be applied to Cp therapy without impeding its therapeutic goals, needs to be evaluated in further studies.

## Figures and Tables

**Table 1:** The primer information in RT-qPCR.

Target Gene	Primer	Sequence (5′–3′)	Product Size (bp)
β-actin	Forward	GTGCTATGTTGCTCTAGACTTCG	174
	Reverse	ATGCCACAGGATTCCATACC	
IFN-γ	Forward	CTGGAGGAACTGGCAAAAGGATGG	121
	Reverse	GACGCTTATGTTGTTGCTGATGGC	
TNF-α	Forward	GGACTAGCCAGGAGGGAGAACAG	103
	Reverse	GCCAGTGAGTGAAAGGGACAGAAC	
p65	Forward	AGACCCAGGAGTGTTCACAGACC	141
	Reverse	GTCACCAGGCGAGTTATAGCTTCAG	
P38	Forward	GGGCATCGTGTGGCAGTTAAGAAG	86
	Reverse	AGCAGACGCAACTCTCGGTAGG	
JNK	Forward	GCCTTATGTGGTGACTCGCTACTAC	104
	Reverse	TTTCTCCCATGATGCACCCAACTG	
IL-2	Forward	GCAGCTCGCATCCTGTGTCAC	97
	Reverse	CTGCTGTGCTTCCGCTGTAGAG	
IL-6	Forward	CTTCTTGGGACTGATGCTGGTGAC	91
	Reverse	TCTGTTGGGAGTGGTATCCTCTGTG	
IL-10	Forward	TGCCAAGCCTTATCGGAAATGATCC	131
	Reverse	AGCCGCATCCTGAGGGTCTTC	

**Table 2:** Effects of JFP-Ps on the content of MDA and activities of SOD, CAT and GSH-Px.

Group	MDA (nmol/g)	SOD (U/g)	CAT (μmol/min/g)	GSH-Px (nmol/min/g)
NC	21.39 ± 0.36	351.18 ± 12.51	1320.94 ± 50.29	5042.16 ± 49.24
MC	25.96 ± 1.08 *	214.84 ± 11.47 **	567.75 ± 75.15 **	3409.86 ± 269.76 **
LG	24.41 ± 0.96	281.60 ± 14.09 **^#^	756.92 ± 54.03 **	4519.30 ± 163.22 ^##^
MG	24.26 ± 0.87	287.17 ± 8.45 *^##^	1131.68 ± 77.32 ^##^	4792.28 ± 78.52 ^##^
HG	22.84 ± 0.96	329.26 ± 17.10 ^##^	1140.23 ± 204.46 ^##^	4951.07 ± 29.22 ^##^

Compared with NC: * *p* < 0.05, ** *p* < 0.01; Compared with MC: ^#^ *p* < 0.05, ^##^ *p* < 0.01.

**Table 3:** Effects of JFP-Ps on the levels of cytokines.

Group	IL-2 (pg/mL)	IL-6 (pg/mL)	TNF-α (pg/mL)	IFN-γ (pg/mL)
NC	281.76 ± 21.34	112.26 ± 1.92	557.32 ± 13.90	496.32 ± 12.34
MC	256.15 ± 7.45	102.49 ± 4.68 *	515.06 ± 24.10	553.22 ± 14.51 *
LG	259.03 ± 6.30	101.91 ± 2.02 *	522.20 ± 21.70	527.93 ± 15.73
MG	271.30 ± 9.25	103.98 ± 1.55	527.56 ± 33.42	500.63 ± 19.57 ^#^
HG	281.61 ± 7.74	106.10 ± 2.55	548.10 ± 18.18	494.60 ± 16.43 ^#^

Compared with NC group: * *p* < 0.05; Compared with MC group: ^#^ *p* < 0.05.

**Table 4:** Results of data quality control using *t*-test.

Mode		*p* All	*p* < 0.05	*p* < 0.02	*p* < 0.01	*p* < 0.005	*p* < 0.001
ESI(+)	corrected *p*-value	966	227	170	137	114	80
	expected by chance	/	11	3	1	0	0
ESI(−)	corrected *p*-value	1614	398	290	244	210	141
	expected by chance	/	19	5	2	1	0

**Table 5:** The significant metabolites in the liver (ESI+).

No.	Rt (min)	*m*/*z*	Identification	Formula	Structure
1	1.294	146.1633	Spermidine	C_7_H_19_N_3_	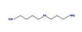
2	1.374	147.1114	L-Lysine	C_6_H_14_N_2_O_2_	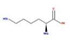
3	1.384	133.0964	Ornithine	C_5_H_12_N_2_O_2_	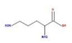
4	1.452	156.0755	L-Histidine	C_6_H_9_N_3_O_2_	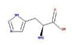
5	1.714	116.0696	L-Proline	C_5_H_9_NO_2_	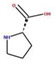
6	2.002	150.0569	L-Methionine	C_5_H_11_NO_2_S	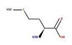
7	2.034	123.0543	Niacinamide	C_6_H_6_N_2_O	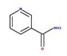
8	2.125	153.0395	Xanthine	C_5_H_4_N_4_O_2_	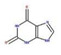
9	2.342	137.0453	Hypoxanthine	C_5_H_4_N_4_O	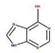
10	2.578	130.0495	N-Acryloylglycine	C_5_H_7_NO_3_	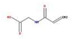
11	2.844	268.1034	2’-Deoxyguanosine	C_10_H_13_N_5_O_4_	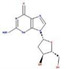
12	3.020	132.1007	L-Isoleucine	C_6_H_13_NO_2_	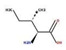
13	3.308	308.0888	Glutathione	C_10_H_17_N_3_O_6_S	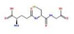
14	3.579	285.0809	Xanthosine	C_10_H_12_N_4_O_6_	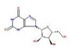
15	4.462	166.0850	L-Phenylalanine	C_9_H_11_NO_2_	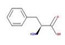
16	5.670	136.0610	Adenine	C_5_H_5_N_5_	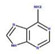
17	5.723	298.0954	5’-Methylthioadenosine	C_11_H_15_N_5_O_3_S	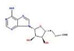
18	5.932	205.0955	L-Tryptophan	C_11_H_12_N_2_O_2_	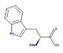
19	5.971	146.0589	Indole-3-carboxaldehyde	C_9_H_7_NO	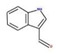
20	13.519	318.2980	Phytosphingosine	C_18_H_39_NO_3_	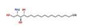
21	15.564	302.3030	Sphinganine	C_18_H_39_NO_2_	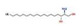
22	16.319	300.2889	Sphingosine	C_18_H_37_NO_2_	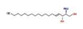
23	29.679	700.5739	Glucosylceramide	C_40_H_77_NO_8_	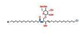

**Table 6:** The significant metabolites in the liver (ESI−).

No.	Rt (min)	*m*/*z*	Identification	Formula	Structure
1	1.411	154.0594	L-Histidine	C_6_H_9_N_3_O_2_	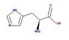
2	1.651	124.0053	Taurine	C_2_H_7_NO_3_S	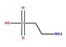
3	1.793	115.0015	Fumaric acid	C_4_H_4_O_4_	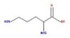
4	1.983	135.0287	Hypoxanthine	C_5_H_4_N_4_O	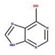
5	2.169	128.0328	Pyroglutamic acid	C_5_H_7_NO_3_	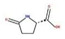
6	2.667	151.0235	Oxypurinol	C_5_H_4_N_4_O_2_	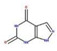
7	2.703	117.0171	Succinic acid	C_4_H_6_O_4_	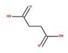
8	2.828	130.0848	L-Norleucine	C_6_H_13_NO_2_	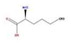

**Table 7:** Metabolite pathway enrichment.

Pathway Name	Match Status	*p*	−log (*p*)	FDR	Impact
Aminoacyl-tRNA biosynthesis	7/48	8.8845 × 10^−6^	5.0514	7.463 × 10^−4^	0.24136
Sphingolipid metabolism	4/21	3.43 × 10^−4^	3.4647	0.014406	0.21875
Purine metabolism	6/66	6.4983 × 10^−4^	3.1872	0.018195	0.0909
Glutathione metabolism	4/28	0.0010804	2.9664	0.022689	0.24325
Arginine biosynthesis	2/14	0.022968	1.6389	0.36155	0.0625
Arginine and proline metabolism	3/38	0.025825	1.588	0.36155	0.125
Citrate cycle (TCA cycle)	2/20	0.045012	1.3467	0.5169	0.06897

## Data Availability

The data sets generated during and/or analyzed during the current study are either shown in the manuscript or are available from the corresponding author on reasonable request.
